# Establishing the Kidney dIsease in the National guarD (KIND) registry: an opportunity for epidemiological and clinical research in Saudi Arabia

**DOI:** 10.1186/s12882-024-03479-0

**Published:** 2024-02-19

**Authors:** Mohammed Tawhari, Moustafa Alhamadh, Abdulrahman Alhabeeb, Abdulaziz Ureeg, Suliman Alghnam, Fayez Alhejaili, Lubna A. Alnasser, Abdullah Sayyari

**Affiliations:** 1 College of Medicine, King Saud Bin Abdulaziz University for Health Sciences, Ministry of National Guard - Health Affairs, Riyadh, Saudi Arabia; 2 Department of Medicine, Division of Nephrology, King Abdulaziz Medical City, Ministry of National Guard - Health Affairs, Riyadh, Saudi Arabia; 3 Population Health Research Section, King Abdullah International Medical Research Center, Riyadh, Saudi Arabia; 4 King Saud bin Abdulaziz University for Health Sciences, Ministry of the National Guard--Health Affairs, Riyadh, Saudi Arabia

**Keywords:** Renal registry, Kidney disease, Renal transplant, Saudi Arabia, Chronic kidney disease

## Abstract

**Background:**

In Saudi Arabia (SA), there has been an alarming increase in the prevalence of chronic kidney diseases (CKD) over the last three decades. Despite being one of the largest countries in the Middle East, renal conditions remain understudied, and there is limited data on their epidemiology and outcomes in SA.

**Objectives:**

To document the experience of establishing a local renal registry assessing the epidemiology of CKD and identifying potential areas for improving the quality and delivery of care for CKD patients.

**Methods:**

This is a multi-center retrospective registry. Potential participants were identified through the ICD-10 codes from five hospitals serving the National Guard affiliates in SA. Patients aged ≥ 18 years treated in any National Guard hospital since 2010 for glomerulonephritis, CKD, or received hemodialysis, peritoneal dialysis, or renal transplant were enrolled. Once enrolled in the registry, patients were followed to the last visit date. RedCap was used to create and host the online registry platform.

**Results:**

A total of 2,912 patients were included, and more than half were younger than 60 years old. Two-thirds of the patients were overweight (25%) or obese (37%). Glomerulonephritis was diagnosed in 10% of the patients, and dialysis-dependent and kidney transplant patients accounted for 31.4% and 24.4%, respectively. Hypertension and diabetes mellitus were detected among 52% and 43% of the participants, respectively. Hemodialysis was the most prevalent dialysis method, with patients spending 3.6 ± 0.4 h per session to receive this treatment. One in every five participants had a kidney biopsy taken (21%). Several barriers and facilitators of the success of this registry were identified.

**Conclusions:**

The KIND registry provides much-needed information about CKD in Saudi Arabia and serves as a model for future projects investigating the natural history and progression of the spectrum of renal diseases. Logistic and financial challenges to the sustainability of registries are identified and discussed.

**Supplementary Information:**

The online version contains supplementary material available at 10.1186/s12882-024-03479-0.

## Introduction

Chronic Kidney Disease (CKD) is a clinical syndrome whose global prevalence is increasing yearly, currently estimated at 13.4% [1]. This increase exerts substantial pressure on healthcare systems, dramatically impacting patients’ quality of life and leading to premature mortality, primarily due to cardiovascular complications [2, 3]. The escalating rate of CKD is mainly attributed to diabetes mellitus (DM), hypertension (HTN), glomerulonephritis, autoimmune diseases, and polycystic kidney disease [4].

End-stage Kidney Disease (ESKD) is a severe stage of CKD, necessitating kidney replacement therapy (KRT) such as dialysis or transplantation [5]. However, the incidence of dialysis-dependent patients continues to surge due to the rising prevalence of metabolic syndrome, DM, HTN, and exposure to environmental toxins in certain regions [6, 7]. While dialysis offers a solution, the mortality rate associated with ESKD remains high, with a five-year survival rate varying between 41%-60% based on the reporting country [8]. Patients with ESKD have a significantly lower quality of life, functional status, and symptom burden than most other chronic conditions, including heart failure, chronic pulmonary disease, and cancer [9, 10]. Therefore, longitudinal studies are critical to evaluate the relationship between risk factors, the development of specific kidney diseases, and the long-term outcomes of different KRTs.

Registries offer a systematic approach to collecting observational data on specific outcomes of a disease- or condition-defined population for scientific, clinical, or policy purposes [11]. They serve as information databases of clinical relevance, such as disease patterns, associated risk factors, clinical care provided, and outcomes of interest. Such data can spotlight potential areas of improvement, fostering enhancements in healthcare delivery [12]. Even with their observational nature, registries facilitate researchers in hypothesis generation and evaluation, thereby furthering scientific progress. They also offer a pool of potential patients for prospective interventional studies [13]. Numerous kidney disease registries have been established globally [14–19] and have proven to be powerful tools to develop best practice guidelines, improve the performance of dialysis centers, and enhance outcomes and cost savings [20, 21].

A few kidney disease databases exist in Saudi Arabia (SA). To begin with, the Saudi Center for Organ Transplantation (SCOT), established around four decades ago, provides annual reports on organ donation and KRT in SA. Based on the latest available report, there is nearly 30,000 patients on KRT in SA [22]. Furthermore, the Gulf Cooperation Council's Dialysis Outcomes and Practice Patterns Study (GCC DOPPS), which involves 20 Saudi centers and 460 randomly chosen dialysis-dependent patients, offers important insights into the clinical and demographic traits of hemodialysis (HD) patients [23]. In addition, a kidney disease registry, involving 782 patients with biopsy-proven primary glomerulonephritis from six major hospitals in SA, was established 23 years ago, but it stopped publishing further work beyond the year 2000 [24].

However, despite being one of the most advanced medical systems in the Middle East, SA lacks a national kidney disease registry, including the different types of kidney disease. Instead, local observational studies have been the primary source of context-specific information on the different types of kidney disease [25–27]. Some of the observational studies suggested that the prevalence of kidney diseases in SA may differ from the other countries [24].

Acknowledging this gap, we initiated a registry for kidney disease patients receiving care at the Ministry of National Guard-Health Affairs. Our goals were three-fold: 1) to assess the burden and distribution of the different forms of kidney disease, 2) to identify the areas for potential quality improvement in terms of access, delivery of care, and patient safety, and 3) to identify areas for research to advance the care for patients with CKD in SA.

This paper summarizes our experience developing the *Kidney dIsease in the National guarD* (KIND) registry in one of the largest national healthcare systems, sheds light on some of the challenges encountered, and shares some key findings of the KIND registry.

## Methods

### Settings

This multi-center registry spans five hospitals serving beneficiaries of the Ministry of National Guard, including King Abdulaziz Medical City (KAMC-R) in Riyadh (1,501 beds), which is the largest site and represents the Central region, and King Abdulaziz Medical City (KAMC-J) in Jeddah (751 beds), representing the Western region. The registry also covers smaller hospitals such as King Abdulaziz Hospital in Al Ahsa (300 beds), Al-Imam Abdulrahman bin Faisal Hospital in Dammam (100 beds), serving the Eastern region, and Prince Mohammad bin Abdulaziz Hospital in Al Madinah (215 beds). The registry started with data collection from KAMC-R for the first year, followed by including other sites.

### Study participants

All patients aged 18 or above were included in this registry, utilizing the unified electronic health record (EHR) system deployed across the National Guard Health Affairs (NGHA) hospitals since 2016. Previous patient medical records from 2010–2016 have been digitalized and integrated into the EHR. A built-in query system was utilized to extract data of patients diagnosed with glomerulonephritis confirmed by renal biopsy, patients with CKD undergoing hemodialysis or peritoneal dialysis, and those who had undergone renal transplantation across all NGHA regions.

Two experienced nurses who served as research coordinators were trained over three sessions to extract data from the EHR. Patients were identified via a combination of ICD-10 codes and keyword search of potential diagnoses within clinical notes. We opted for this combination for two reasons: i) to capture patients diagnosed or treated pre-2016 through searching digitalized paper-based records when ICD-10 codes were not used in the paper-based system, and ii) to address the potential inconsistent adoption of ICD-10 codes designation when electronic medical records were first introduced in 2016. Then, the research coordinators verified the diagnoses, identified patients for registry inclusion, abstracted patient information, and input data into an electronic form. The online registry platform was created and hosted using REDCap® platform.

For data validation, the principal investigator, a nephrology consultant proficient in EHR system use, conducted training sessions for the research coordinators. Regular meetings were held to address questions and ensure standardization of the data entry process. To further ensure efficient communication, the coordinators were chosen from KAMC-R. Additionally, an independent data registrar conducted periodic checks of randomly selected data samples, amounting to 1% of the total data annually—any detected discrepancies led to meetings with the principal investigator for resolution and guidance for future improvements. The primary discrepancy source was data scanned from handwritten reports into the EHR.

After inclusion in the registry, each patient was followed up to their last available visit. This allowed tracking the patient’s journey, from initial CKD diagnosis to their most recent health status. For instance, a patient initially diagnosed with glomerulonephritis could transition to peritoneal dialysis, then to hemodialysis, receive a transplant, and potentially return to dialysis. If patients received treatment at different hospitals within the National Guard Health system, their records were linked via the unique identifiers.

The data elements in the registry were grouped into six sections (Table 1). First, we captured general information, including demographics, medical history, and the body mass index (BMI) at diagnosis. Second, we captured information related to glomerulonephritis, including the vital signs, creatinine levels, and other laboratory measurements at the date of diagnosis and subsequently every six months afterwards. Third, we collected information related to renal transplantation, including the source of the kidney and the procedure-related data. Fourth, we captured the dialysis type and modality for patients undergoing dialysis. Fifth, we captured the hematology, urine, biochemistry, immunology, and other laboratory measurements collected at the time of diagnosis and every six months. In addition, the data registrar extracted information to determine if the patient underwent a radiology ultrasound and/or biopsy. Lastly, according to the latest follow-up, we captured whether the patient is still alive. The reporting of this study follows the Strengthening the Reporting of Observational studies in Epidemiology (STROBE) guidelines.

**Table 1:** Scope of data collected for the KIND registry

**First section: Demographic/ anthropometric/ medical history characteristics**
Date of birth, sex, occupation, region within Saudi Arabia, height, weight, and Body mass index (BMI). Medical history included: status of diabetes mellitus, hypertension, and coronary artery disease
**Second section: Glomerulonephritis patients**
Biopsy diagnosis, history of symptoms, systolic and diastolic blood pressure, urine analysis, creatinine, glomerular filtration rate (GFR), Albumin-to-Creatinine ratio (ACR), Protein-Creatinine Ratio (PCR), 24 h protein, Serum Albumin, Anti–glomerular basement membrane (anti-GBM), Antineutrophil cytoplasmic autoantibodies (ANCA), Antinuclear antibodies (ANA), Anti-double stranded DNA (Anti-dsDNA), Complement levels (C3&C4), Viral serology (HBV, HCV and HIV), Cryoglobulin level, erythrocyte sedimentation rate (ESR), Rheumatoid Factor (RF), Hematuria, family history of Glomerulonephritis, and medication history
**Third section: Transplanted patients**
Details of the transplant, hourly urine at the date of transplant, kidney function indicators after transplant, hospitalization, infections, the occurrence of malignancies, cardiovascular diseases, or pregnancy
**Fourth section: Patients on dialysis**
History of dialysis treatment, frequency and duration of dialysis sessions, eGFR, type of HD method (high/low flux), type of peritoneal dialysis (continuous ambulatory or cyclic PD, nocturnal intermittent PD), Urea Reduction Ratio, Kt/V rate, Residual renal function, organisms isolated during dialysis
**Fifth section: Laboratory measurements**
White blood cells (WBC), Hemoglobin, Platelets, Serum Iron, Ferritin, Urine RBC, Urine Pus, Urine protein, Urine PCR, Fasting Blood sugar, Urea, Potassium, Sodium, corrected Calcium, Phosphate, Parathyroid hormone (PTH), C-Reactive Protein, Ultra-Sound information: size and echogenicity of kidneys
**Sixth section: Follow-up data**
Patient status, cause of death, loss-to-follow-up
**Keywords used to identify patients:** Glomerulonephritis, Hemodialysis, Peritoneal dialysis, Renal transplant, Kidney transplant, chronic kidney disease

### Statistical analysis

Statistical analyses were performed using STATA version 15.0 software. The categorical data are presented as frequencies and percentages, and the continuous data as means and standard deviation, or medians and interquartile range (IQR). We stratified results by sex to examine any potential differences in the distribution of population characteristics. Percentage of missing data, if any, are presented for each variable; and often represented data that was not captured in EHR or not readily identifiable from paper-based records.

### Ethical considerations

The Institutional Review Board (IRB) at the King Abdullah International Medical Research Center (KAIMRC) reviewed, approved, and funded the study, with protocol number 20–419812-54,071.

## Results

The KIND registry encompasses a total of 2,912 patients. More than half of these patients (55.5%) were under the age of 60, and 13.7% were under 30 years of age (Table 2). A significant majority of the participants, 64%, were overweight or obese. Data showed that 73% of the participants were unemployed, and most patients were identified from the central region (Table 2).

**Table 2:** Socio-demographic characteristics of the participants in the KIND registry (*n* = 2,912)

Variable	Male	Female	Total
			**N (%)**
**Age**			
≤ 30 years	226 (13.7)	172 (13.6)	398 (13.7)
31–40	225 (13.7)	127 (10.0)	352 (12.1)
41–50	223 (13.5)	176 (13.9)	399 (13.7)
51–60	244 (14.8)	223 (17.6)	467 (16.0)
> 61	729 (44.3)	567 (44.8)	1,296 (44.5)
Mean age (± SD)	53.88 (20.3)	54.10 (19.4)	53.98 (19.9)
**Region**			
Central	1,317 (80.0)	1,007 (79.6)	2,324 (79.8)
Eastern	110 (6.7)	120 (9.5)	230 (7.9)
Western	178 (10.8)	109 (8.6)	287 (9.9)
Madinah	30 (1.8)	20 (1.6)	50 (1.7)
Missing	12 (0.7)	9 (0.7)	21 (0.7)
**Nationality**			
Saudi	1,545 (93.8)	1,190 (94.1)	2,735 (93.9)
Non-Saudi	82 (5.0)	51 (4.0)	133 (4.6)
Missing	20 (1.2)	24 (1.9)	44 (1.5)
**Eligibility**			
NGHA Eligibility	894 (54.3)	777 (61.4)	1,671 (57.4)
Disease-based eligibility	600 (36.4)	368 (29.1)	968 (33.2)
Missing	153 (9.3)	120 (9.5)	273 (9.4)
**Marital Status**			
Single	429 (26.1)	254 (20.1)	683 (23.5)
Married	1,065 (64.7)	707 (55.9)	1,772 (60.9)
Divorced	6 (0.4)	54 (4.3)	60 (2.1)
Widowed	7 (0.4)	162 (12.8)	169 (5.8)
Missing	140 (8.5)	88 (7.0)	228 (7.8)
**Occupation**			
Employed	106 (6.4)	19 (1.5)	125 (4.3)
Retired	11 (0.7)	1 (0.1)	12 (0.4)
Unemployed	1,079 (65.5)	1,037 (82.0)	2,116 (72.7)
Student	32 (1.9)	30 (2.4)	62 (2.1)
Child	75 (4.6)	56 (4.4)	131 (4.5)
Missing	344 (20.9)	122 (9.6)	466 (16.0)
**Body Mass index (BMI)**			
< 18.5	155 (9.4)	104 (8.2)	259 (8.9)
18.5–24.9	480 (29.1)	305 (24.1)	785 (27.0)
25–29.9	481 (29.2)	301 (23.8)	782 (26.9)
≥ 30	525 (31.9)	555 (43.9)	1,080 (37.1)
Missing BMI data	6 (0.4)	0 (0.0)	6 (0.2)
Mean BMI (± SD)	27.16 (7.4)	29.03 (8.5)	27.98 (7.9)

Over half of the patients (51.9%) were hypertensive, and more than a third (42.8%) had DM (Table 3). The most frequent cause of CKD was diabetic nephropathy, followed by glomerulonephritis and hypertensive nephropathy, accounting for 35.2%, 17.2%, and 12.7%, respectively. Approximately one-quarter of the registry participants were found to have CKD with unknown causes, while just under 10% exhibited CKD attributed to multiple factors. Polycystic kidney disease, reflux nephropathy, pyelonephritis, and stones collectively accounted for less than 1%, contributing to a minority of CKD cases within the KIND registry (Table 3).

**Table 3:** Clinical characteristics of participants in the KIND registry (*n* = 2,912)

Variable	Male	Female	Total
	**N (%)**		
**Diabetes Mellitus (ICD10: E10, E11, and E13)**			
No	962 (58.4)	703 (55.6)	1,665 (57.2)
Yes	685 (41.6)	562 (44.4)	1,247 (42.8)
**Hypertension (ICD10: I10-I15)**			
No	802 (48.7)	598 (47.3)	1,400 (48.1)
Yes	845 (51.3)	667 (52.7)	1,512 (51.9)
**Coronary artery disease (ICD10: I20-I25)**			
No	1,591 (96.6)	1,242 (98.2)	2,833 (97.3)
Yes	56 (3.4)	23 (1.8)	79 (2.7)
**Smoking status**			
No	266 (16.2)	273 (21.6)	539 (18.5)
Yes	10 (0.6)	2 (0.2)	12 (0.4)
Unknown	1,371 (83.2)	990 (78.3)	2,361 (81.1)
**Classification of patients (based on kidney disease or KRT)**			
Glomerulonephritis	163 (9.9)	131 (10.4)	294 (10.1)
ESKD on dialysis	449 (27.3)	466 (36.8)	915 (31.4)
ESKD Post-transplant	441 (26.8)	278 (22.0)	719 (24.7)
More than one type^a^	594 (36.1)	390 (30.8)	984 (33.8)
Mean age (in years) at onset of symptoms (SD)	37.32 (17.6)	37.88 (18.3)	37.55 (17.9)
Mean age (in years) at biopsy (SD)	38.25 (17.7)	38.23 (18.2)	38.24 (17.9)
**Cause of CKD**			
Diabetic Nephropathy (ICD-10: E1021, E1022, E1121, E1122, E1322)	559 (33.9)	466 (36.8)	1,025 (35.2)
Glomerulonephritis (ICD10: N50-N59)	288 (17.5)	214 (16.9)	502 (17.2)
Hypertensive renal disease (ICD-10: I120, I129-I132)	219 (13.3)	150 (11.9)	369 (12.7)
Pyelonephritis (ICD10: N10, N12, and N119)	6 (0.4)	6 (0.5)	12 (0.4)
Polycystic kidney disease (ICD10: Q613)	8 (0.5)	2 (0.2)	10 (0.3)
Reflux nephropathy (ICD-10: N137)	4 (0.2)	6 (0.5)	10 (0.3)
Calculi (ICD-10: N20.0 and N200)	7 (0.4)	2 (0.2)	9 (0.3)
Unknown	407 (24.7)	305 (24.1)	712 (24.5)
Others (ICD10: N12, N170, I129, Q611, and M1036)	7 (0.4)	9 (0.7)	16 (0.6)
Multiple causes from those listed above	142 (8.6)	105 (8.3)	247 (8.5)
**Biopsy details**			
No biopsy taken	1,299 (78.9)	1,026 (81.1)	2,325 (79.8)
Biopsy was taken at NGHA	348 (21.1)	239 (18.9)	587 (20.2)
**Family History of Glomerulonephritis**			
No	328 (19.9)	231 (18.3)	559 (19.2)
Yes	7 (0.4)	5 (0.4)	12 (0.4)
Unknown	1,312 (79.7)	1,029 (81.3)	2,341 (80.4)
**Dialysis History**			
**Ever on dialysis**			
No	648 (39.3)	435 (34.4)	1,083 (37.2)
Yes	999 (60.7)	830 (65.6)	1,829 (62.8)
**Type of initial dialysis modality (if ever on dialysis)**			
Hemodialysis (HD)	924 (92.5)	730 (88.0)	1,654 (90.4)
Peritoneal dialysis (PD)	61 (6.1)	83 (10.0)	144 (7.9)
Hemodiafiltration	0 (0.0)	2 (0.2)	2 (0.1)
Missing	14 (1.4)	15 (1.8)	29 (1.6)
**Type of hemodialysis**			
High flux	518 (56.1)	490 (67.1)	1,008 (60.9)
Low flux	3 (0.3)	3 (0.4)	6 (0.4)
Missing	403 (43.6)	237 (32.5)	640 (38.7)
**Type of Peritoneal dialysis**			
Continuous ambulatory PD	5 (8.2)	8 (9.6)	13 (9.0)
Continuous cyclic PD	1 (1.6)	9 (10.8)	10 (6.9)
Nocturnal intermittent PD	1 (1.6)	2 (2.4)	3 (2.1)
Missing	54 (88.5)	64 (77.1)	118 (81.9)

*CKD* Chronic Kidney Disease, *ESKD* End-stage kidney disease, *ICD-10*: International classification of diseases-10th revision, *KRT* Kidney Replacement Therapy, *NGHA* National Guard Health Affairs, *SD* standard deviation

^a^This category includes participants who had data on more than one type of kidney disease throughout the study period. For example, a Glomerulonephritis patient who received Peritoneal dialysis and then switched to HD

Concerning the type of kidney disease, 10.1% of the patients were diagnosed with glomerulonephritis, 31.4% were undergoing dialysis, and 24.4% had undergone a kidney transplant (Table 3). Approximately one-third of the patients (33.8%) had information on at least two out of the three stages of kidney disease or KRT. This suggests these patients had progressed from one stage to another during the observation period. Furthermore, one in every five participants had a biopsy performed at an NGHA facility (Table 3).

Supplementary Table 1 presents the histopathologic findings from kidney biopsies. Almost two-thirds of the patients who underwent kidney biopsy showed evidence of glomerulonephritis (57.24%). Within this category, membranoproliferative glomerulonephritis and segmental glomerulosclerosis accounted for 25.7% and 20% of all biopsies. The most prevalent finding among transplant biopsies was acute T-cell mediated rejection. Tubulointerstitial kidney disease and diabetic kidney disease were detected in 13.6% and 8.2% of all biopsies. In terms of dialysis, hemodialysis (HD) was the most prevalent method; it was observed that 90% of participants who needed dialysis began with HD (Table 3). Of note, NGHA hospitals predominantly employ high-flux HD filters.

The initial and 6-month follow-up laboratory results, stratified by kidney disease type or KRT, are displayed in Table 4. It should be noted that this table serves the primary purpose of providing an overview of the extensive data captured by the KIND registry and is not intended for direct comparison due to the distinct characteristics of each disease or KRT type.

**Table 4:** Selected laboratory results for participants in the KIND registry by kidney disease type (*n* = 2,912)

Variable	Type of kidney disease or Kidney Replacement Therapy (KRT)			
	**Glomerulonephritis**	**Hemodialysis**	**Peritoneal dialysis**	**Transplant**
	**Median (IQR)**			
**Systolic blood pressure (mmHg)**				
Initial value	135 (123 – 148)	138 (127 – 151)	132.5 (121.5—148.5	136 (124 – 148)
6 months later	132 (122 – 146)	135 (124—147)	131.5 (115 -149.5)	131 (122—145)
**Diastolic blood pressure (mmHg)**				
Initial value	77 (69—85)	78.5 (69—86)	76.5 (70—89)	78 (70 – 86)
6 months later	75 (66—83)	75 (66—84)	78 (69.5—87.5)	76 (67 – 84)
**eGFR (mL/min/1.73m2)**				
Initial value	33.5 (15—53)	28 (12—49)	19 (10—50)	34 (17—51)
6 months later	36 (15 – 60)	26 (11—53)	16.5 (8—36)	37 (15.5 -57)
**Serum creatinine (U/L)**				
Initial value	173 (108—331)	219 (144—438)	205 (120—370)	179 (129—329)
6 months later	163 (100 – 348)	229 (126—493)	352.5 (156—649)	178.5 (114.5 -395.5)
**Serum albumin g/L**				
Initial value	34 (28 – 38)	34 (29—38)	35 (28—38)	36 (31—40)
6 months later	38 (34 – 42)	38 (34 – 42)	37 (33—43)	41 (35—43)
**ESR (mm/hr)**				
Initial value	48 (24—77)	49 (21—76)	80 (30—120)	33.5 (16 – 70)
6 months later	40 (24 – 66)	40 (25—77)	24 (19—55)	40 (24—79)
**PCR (mg/mg)**				
Initial value	5.3 (1.5 -14.9)	4.8 (0.9—12.5)	1.2 (0.5—2.3)	3.1 (0.5—12.2)
6 months later	3.0 (1.1—12.9)	3.2 (1.2 – 10.0)	0.7 (0.2—1.1)	2.1 (0.4—8.7)
**Urea reduction ratio (%)**				
Initial value		73 (68—78)		
6 months later		74 (69—79)		
**Hemoglobin (g/L)**				
Initial value	109 (92—127)	100 (86—116.5)	100 (87—116)	107 (93 – 123)
6 months later	126 (112 – 139)	121 (107 – 135)	114 (100 – 131)	135 (120 – 148)
**Corrected calcium (mmol/L)**				
Initial value	2.3 (2.2—2.4)	2.2 (2.1—2.3)	2.3 (2.1—2.4)	2.2 (2.1—2.4)
6 months later	2.3 (2.2—2.4)	2.3 (2.2—2.4)	2.3 (2.2—2.4)	2.3 (2.3—2.4)
**Phosphorus (mmol/L)**				
Initial value	1.4 (1.1—1.8)	1.5 (1.2—2.0)	1.7 (1.3—2.0)	1.4 (1.1—1.8)
6 months later	1.3 (1.1—1.5)	1.2 (1.0—1.5)	1.3 (1.1—1.7)	1.2 (1.0—1.3)
**Potassium (mmol/L)**				
Initial value	4.3 (4.0—4.8)	4.4 (4.0—5.0)	4.3 (3.8—4.8)	4.3 (3.9—4.9)
6 months later	4.4 (4.0—4.8)	4.5 (4.1—5.0)	4.4 (3.9—4.8)	4.3 (4.0—4.6)
**Complement Component 3 (G/L)**				
Initial value	1.1 (0.9–1.3)			
6 months later	1.0 (0.8–1.1)			
**Complement Component 4 (G/L)**				
Initial value	0.3 (0.2–0.4)			
6 months later	0.3 (0.2–0.4)			
**Anti-DNA (IU/mL)**				
Initial value	16.0 (8.0–31.0)			
6 months later	15.6 (7.2–34.8)			
**Perinuclear anti-neutrophil cytoplasmic antibodies (p-ANCA) (U/mL)**				
Initial value	2.1 (1.4–3.7)			
6 months later	2.5 (1.4–7.0)			
**cytoplasmic anti-neutrophil cytoplasmic antibody (cANCA) (U/mL)**				
Initial value	2.3 (1.8–3.5)			
6 months later	2.1 (1.3–2.6)			
**Anti-glomerular basement membrane (Anti-GBM) (U/mL)**				
Initial value	1.2 (0.7–2.0)			
6 months later	0.7 (0.3–7.8)			
**Parathyroid Hormone (PTH) (pmol/L)**				
Initial value	46.6 (21.5–97.6)			
6 months later	31.3 (13.6–85.7)			
**Hepatitis B surface antigen (HBsAg) (%)**				
Negative	476 (62.6)			
Positive	12 (1.6)			
Unknown	273 (35.9)			
**Hepatitis C (HCV) (%)**				
Negative	445 (58.5)			
Positive	28 (3.7)			
Unknown	288 (37.8)			
**Human immunodeficiency virus (HIV) (%)**				
Negative	22 (2.9)			
Positive	1 (0.1)			
Unknown	738 (97.0)			
**Dialysis details**				
Average number of dialysis sessions/ week (± SD)		3.0 (0.2)		
Average number of hours/ dialysis session (± SD)		3.6 (0.4)		

Initial value refers to laboratory results closest to the diagnosis date ± 3 months, while the 6-month follow up summarizes laboratory results for 6 months following the initial value

For patients with glomerulonephritis, the median estimated glomerular filtration rate (eGFR), using the 2009 CKD-EPI equation, at the initial assessment was 33.5 (IQR: 15—53) mL/min/1.73 m^2^. Among participants on HD, the median albumin level was 34 g/L (IQR: 29–38), and the median urea reduction rate was 73% (IQR: 68–78%) (Table 4). The median number of weekly HD sessions was 3.0 ± 0.2, with each session lasting an average of 3.6 ± 0.4 h (Table 4).

## Discussion

The KIND registry, currently the largest CKD registry in SA, presents an invaluable source for future research and a blueprint for subsequent local and national registries. However, the registry’s development was not without difficulties, and by acknowledging these challenges, we hope to inform the creation of future registries. Before delving into the challenges of establishing the KIND registry, we would like to highlight a few key observations.

The first observation is the relatively high unemployment rate (73%) among KIND registry participants. Although this rate is higher than what has been reported in the literature, the low employment rate has been observed by local and international reports [28, 29]. For example, in the United States, based on recent reports, almost two-thirds of the patients are either unemployed or retired, with only 11.3% employed full- or part-time [28]. Furthermore, based on the GCC DOPPS report, only 28% of the participants were either full- or part-time employed [23]. The relatively higher unemployment rate among our patients can be attributed to several reasons. First, the different categories of employment status that were reported in each study [23, 28]. In the KIND registry, homemakers, which constitute 22% of the GCC DOPPS participants [23], are likely included under “unemployed,” as the Electronic health records we used do not capture this distinction. Thus, the percentage of unemployment in our study is inflated and is higher than in the reported literature. Second, since the NGHA is a military hospital system, we believe that a considerable fraction of our patients are military personnel, and some of the patients could have applied for early retirement due to being on dialysis. Third, the medical records may not have accurate employment records. When we stratified unemployment by age groups (data not shown), half of all unemployed patients were in the 60 + age group, indicating that some could be actually retired, but the EHR did not capture this accurately. Collectively, these results highlight important areas that require further exploration in i) accurately capturing social determinants of health and ii) ensuring that CKD patients remain active for as long as possible.

The second observation is that over two-thirds of our patients were overweight or obese, similar to findings elsewhere. The median BMI was 29.5 kg/m^2^ in a recent report from the US [28]. Moreover, in the GCC DOPPS latest report [23], the median BMI was 26.3 kg/m^2^, which is consistent with our results. The association between obesity and CKD is well established—as obesity is an inflammatory process that causes glomerular and tubular injuries, thus directly and indirectly increasing the risk of Diabetes and Hypertension [30]. Our observations on BMI call for further studies to evaluate and modify the relationship between metabolic syndrome and CKD among the Saudi population.

The third interesting observation in the present study is the relatively young age at the time of symptom onset and renal biopsy, 37.5 and 38.2 years, respectively. The observed average age is younger than some international reports but not much different than previous local literature [31–33]. For example, in a retrospective study evaluating the demographics of patients with ESKD in SA, almost half (40%) of the participants were in the age group of 40–59 years, with nearly one in every four patients (27.8%) being ≥ 60 years old [34]. Likewise, based on the SCOT report, three-quarters (74.1%) of dialysis-dependent patients in the SA are aged ≤ 65 years, the majority (42.7%) of whom fall within the age group of 26–55 years [22]. Similarly, based on the GCC DOPPS, the mean age of dialysis-dependent patients in the Gulf region is 53.2 years [23]. We speculate that this trend of younger patients may be associated with the concerning rise in obesity and DM, contributing to an increased prevalence of diabetic nephropathy [29]. From recent surveys, it is estimated that nearly 25% of the Saudi population, including the young population, is considered obese, thus placing a considerable proportion of Saudis at higher risk of CKD [35]. The younger age at biopsy may reflect local clinical practices where younger individuals are more likely to undergo a biopsy procedure since the risk of autoimmune diseases, including glomerulonephritis, is higher among this age group. Therefore, the biopsy’s yield is more likely to be clinically significant and might change the management plan in the younger group. Ultimately, this interplay between the local burden of obesity, younger age, and CKD onset in Saudi Arabia necessitates exploration of the local and regional profile of patients with CKD to provide policies and public health interventions that are effective and efficient.

The fourth observation is that almost half (42.8%) of the patients were diabetic, with diabetic kidney disease (DKD) being the most frequent cause of CKD. This agrees with both local and international reports. For instance, based on the latest SCOT report, 42% of ESKD causes are attributed to DKD [22]. Likewise, based on the Australia and New Zealand Dialysis and Transplant registry (ANZDATA) and the Chronic Kidney Disease, Queensland (CKD.QLD) registry, DKD was the most frequent cause of CKD, accounting for 40% and 26.7% of the causes, respectively [36, 37]. It should also be noted that almost a quarter of our patients had CKD for unknown reasons, which may just be related to late presentation of CKD when further investigations, including kidney biopsy, would not alter the management. Further probing into this subgroup of patients to assess their access to care is warranted.

The fifth observation is that our participants' biopsy results showed that MPGN and FSGS are the most common glomerular pathologies. This agrees with historical observations made by a Saudi kidney disease registry that included 782 patients from six major hospitals with biopsy-proven primary glomerulonephritis [24].

After examining the key observations of the registry, we pivot our attention to some challenges encountered during establishing the KIND registry. One primary challenge involved the selection of research coordinators. While these individuals were seasoned nephrology nurses, the data collection necessitated specialized data extraction abilities, including reading kidney biopsies and radiological reports and familiarity with the data hosting registry platform. Frequent quality checks by the data registrar and the principal investigator’s accessibility were instrumental in the success of our data collection. Additionally, the health records’ composite nature—EHR and scanned paper-based records—posed another challenge. Although EHR facilitated data location and extraction, extracting information from handwritten documents of varying legibility was more challenging. Lastly, our choice to identify CKD through established diagnoses rather than abnormal laboratory results, as conducted elsewhere, was two-folded [38]. First, this method identifies patients with the highest risk for morbidity and mortality from CKD; therefore, clinical and research resources can prioritize this segment of the population served by the NGHA system. Second, many patients are given disease-based eligibility in NGHA, such as advanced CKD; thus, their abnormal laboratory results indicating earlier stages of CKD are not captured by our healthcare system, which might have underestimated the prevalence of undiagnosed renal diseases. However, given the nature and symptomatology of CKD, it is unlikely we have missed patients with CKD.

Furthermore, the KIND registry’s multi-center nature posed logistic obstacles. Each participating site required separate EHR access, necessitating approval from different entities to facilitate data access from outside the Central region. Discrepancies in practices and diagnostic workups were noted due to the multi-center design involving five hospitals in different cities. For example, some physicians preferred the albumin/creatinine ratio, while others used the protein/creatinine ratio. Moreover, laboratory results were not standardized across sites, necessitating the development of equations to convert results into unified units of measurement. The registry’s observational and retrospective nature also led to sporadic missing data and loss of follow-up for some patients. In addition, patients may get admitted outside of NGHA hospitals. Such patients usually present to the NGHA system later with discharge reports of varying qualities, which increases the risk of missing data. However, as we move forward with our registry, we plan to hire more coordinators to facilitate acquiring records from outside NGHA hospitals.

Funding is a critical factor for the success and continuity of any registry. The KIND registry must persistently gather information to generate longitudinal data invaluable for research and healthcare delivery, demanding sustained engagement from the entire team. Maintaining this engagement and continuous data collection without proper remuneration was challenging for KIND when the funding was consumed. Efforts for securing additional funds are underway to support future activities of the KIND registry.

Despite these hurdles, the KIND registry’s strengths are numerous. It includes 2,912 patients with diverse renal conditions, providing a comprehensive sample size to study CKD’s natural history, evaluate different interventions, and assess prognoses of various conditions. Furthermore, the KIND registry provides valuable data for healthcare system planning, policy decisions, and identifying healthcare delivery gaps. For example, the higher prevalence of overweight/obesity among our patients warrants further research and interventions to comprehend the impact of weight on CKD. Another strength is that a third of the patients transitioned from one CKD stage to another, offering the opportunity to study the effect of early-stage interventions on disease trajectory. Moreover, the registry’s multi-center design increased the sample size, captured geographical variations in CKD prevalence and care delivery, and enhanced the sample’s representativeness. While the current sample size is significant, it is comparatively smaller than some global registries [21, 28, 38]. This is probably due to the fact that in a review of CKD in SA, 65.9% of dialysis-dependent patients were found to be treated by the Ministry of Health hospitals [29]. Still, the multi-center nature of our registry involving five hospitals from different regions of SA, inclusive of national guard employees and their dependents who are not military personnel, coupled with the unique eligibility exception for CKD by NGHA, leads us to believe that the socio-demographic and medical characteristics of our patients are largely reflective of the Saudi population. Moreover, we believe the KIND registry could serve as a nucleus for a more expansive national registry to examine CKD nationally.

In conclusion, despite the above challenges, the KIND registry is a critical step in addressing the increasing CKD burden in SA and the Eastern Mediterranean region. It provides an overview of the current CKD state in SA and serves as a rich data source for future research spanning the entire kidney disease spectrum. To better understand CKD’s burden and trajectory, further efforts should be made to expand the KIND registry to include other healthcare systems in the country, eventually establishing a national CKD registry.

### Supplementary Information

**Additional file 1: Supplementary Table 1.** Results of renal biopsy if biopsy procedure was performed (*n*=587).

## Data Availability

The dataset analyzed during the current study is available from the corresponding author’s institution upon reasonable request. Data inquiries should be directed to researchoffice@mngha.med.sa.

## References

[1] Gaitonde DY, Cook DL, Rivera IM (2017). Chronic kidney disease: detection and evaluation. Am Fam Physician.

[2] Ammirati AL (2020). Chronic Kidney Disease. Rev Assoc Med Bras (1992)..

[3] Lv JC, Zhang LX (2019). Prevalence and disease burden of chronic kidney disease. Adv Exp Med Biol.

[4] Chronic Kidney Disease Prognosis Consortium; Matsushita K, van der Velde M, Astor BC, Woodward M, Levey AS, de Jong PE, et al. Association of estimated glomerular filtration rate and albuminuria with all-cause and cardiovascular mortality in general population cohorts: a collaborative meta-analysis. Lancet. 2010;375(9731):2073–81. Epub 2010 May 17. 10.1016/S0140-6736(10)60674-5 PMC3993088 20483451

[5] Molaoa TT, Bisiwe FB, Ndlovu KC (2021). End-stage kidney disease and rationing of kidney replacement therapy in the free state province, South Africa: a retrospective study. BMC Nephrol.

[6] Himmelfarb J, Vanholder R, Mehrotra R, Tonelli M (2020). The current and future landscape of dialysis. Nat Rev Nephrol.

[7] White Y, Fitzpatrick G. Dialysis: prolonging life or prolonging dying? Ethical, legal and professional considerations for end of life decision making. EDTNA ERCA J. 2006;32(2):99–103. 10.1111/j.1755-6686.2006.tb00460.x 16898103

[8] Thurlow JS, Joshi M, Yan G, Norris KC, Agodoa LY, Yuan CM (2021). Global epidemiology of end-stage kidney disease and disparities in kidney replacement therapy. Am J Nephrol.

[9] Mittal SK, Ahern L, Flaster E, Maesaka JK, Fishbane S (2001). Self-assessed physical and mental function of haemodialysis patients. Nephrol Dial Transplant.

[10] Murtagh FE, Addington-Hall J, Higginson IJ (2007). The prevalence of symptoms in end-stage renal disease: a systematic review. Adv Chronic Kidney Dis.

[11] Gliklich RE, Dreyer NA, Leavy MB, editors. Registries for Evaluating Patient Outcomes: A User's Guide [Internet]. 3rd ed. Rockville (MD): Agency for Healthcare Research and Quality (US); 2014 Apr. Report No.: 13(14)-EHC111. 24945055

[12] Breckenridge K, Bekker HL, Gibbons E, van der Veer SN, Abbott D, Briançon S (2015). How to routinely collect data on patient-reported outcome and experience measures in renal registries in Europe: an expert consensus meeting. Nephrol Dial Transplant.

[13] Hinojosa JA, Pandya AG (2017). The Importance of Patient Registries in Skin of Color. J Investig Dermatol Symp Proc.

[14] Will EJ. A short cultural history of the UK renal registry 1995–2020. BMC Nephrology. 2020;21(1). 10.1186/s12882-020-01997-1 PMC7425145 32787793

[15] Danzig MR, Chang P, Wagner AA, Allaf ME, McKiernan JM, Pierorazio PM (2016). Active surveillance for small renal masses: a review of the aims and preliminary results of the DISSRM Registry. Curr Urol Rep.

[16] Xu H, Lindholm B, Lundström UH, Heimbürger O, Stendahl M, Rydell H (2021). Treatment practices and outcomes in incident peritoneal dialysis patients: the Swedish Renal Registry 2006–2015. Clin Kidney J.

[17] Burke JP, Aljishi M, Francis L, Hoy W, Divi D, Cherian R (2020). Protocol and establishment of a Queensland renal biopsy registry in Australia. BMC Nephrol.

[18] Ozeki T, Maruyama S, Nagata M, Shimizu A, Sugiyama H, Sato H (2020). Committee for Renal Biopsy and Disease Registry of the Japanese Society of Nephrology. The revised version 2018 of the nationwide web-based registry system for kidney diseases in Japan: Japan Renal Biopsy Registry and Japan Kidney Disease Registry. Clin Exp Nephrol..

[19] Martín Escobar E; Registro Español de Enfermos Renales (REER). The Spanish Renal Registry: 2013 report and evolution from 2007–2013. Nefrologia. 2016;36(2):97–120. English, Spanish. Epub 2016 Feb 16. 10.1016/j.nefro.2015.10.020 26895749

[20] Sophia Wong, Catalin Taraboanta, Gordon Francis, Andrew Ignaszewski, Jiri Frohlich. The British Columbia Familial Hypercholesterolemia Registry. BCMJ,2013;55(7):326–330 - Clinical Articles.

[21] Evans KM, Pyart R, Steenkamp R, Caskey FJ (2018). The UK Renal Registry: making patient data matter. Br J Hosp Med (Lond).

[22] Al Attar B. Renal Replacement Therapy in the Kingdom of Saudi Arabia. Saudi J Kidney Dis Transpl. 2021;32(4):1188–1200. 10.4103/1319-2442.338300 35229826

[23] Abouchacra S, Obaidli A, Al-Ghamdi SM (2016). Gulf Cooperation Council-dialysis outcomes and practice patterns study: An overview of anemia management trends at the regional and country specific levels in the Gulf Cooperation Council countries. Saudi J Kidney Dis Transpl.

[24] Huraib S, Al Khader A, Shaheen FA, Abu Aisha H, Souqiyyeh MZ, Al Mohana F, et al. The spectrum of glomerulonephritis in saudi arabia: the results of the saudi registry. Saudi J Kidney Dis Transpl. 2000;11(3):434–41. 18209336

[25] Tawhari M, Alhamadh MS, Alhabeeb A, Almutlaq M, Radwi M (2022). Renal transplant experience in a tertiary care center in Saudi Arabia: a retrospective cohort study. Cureus.

[26] El Minshawy O, Ghabrah T, El Bassuoni E (2014). End-stage renal disease in Tabuk Area, Saudi Arabia: an epidemiological study. Saudi J Kidney Dis Transpl.

[27] Oudah N, Al Duhailib Z, Alsaad K, Qurashi S, Ghamdi G, Flaiw A (2012). Glomerulonephritis with crescents among adult Saudi patients outcome and its predictors. Clin Exp Med.

[28] United States Renal Data System. 2022 USRDS Annual Data Report: Epidemiology of kidney disease in the United States. National Institutes of Health, National Institute of Diabetes and Digestive and Kidney Diseases, Bethesda, MD, 2022.

[29] Al-Sayyari AA, Shaheen FA. End stage chronic kidney disease in Saudi Arabia. A rapidly changing scene. Saudi Med J. 2011;32(4):339–46. 21483990

[30] Wang M, Wang Z, Chen Y, Dong Y (2022). Kidney damage caused by obesity and its feasible treatment drugs. Int J Mol Sci.

[31] Bonani M, Seeger H, Weber N, Lorenzen JM, Wüthrich RP, Kistler AD (2021). Safety of kidney biopsy when performed as an outpatient procedure. Kidney Blood Press Res.

[32] Emelianova D, Prikis M, Morris CS, Gibson PC, Solomon R, Scriver G (2022). The evolution of performing a kidney biopsy: a single center experience comparing native and transplant kidney biopsies performed by interventional radiologists and nephrologists. BMC Nephrol.

[33] Aaltonen S, Finne P, Honkanen E (2020). Outpatient kidney biopsy: a single center experience and review of literature. Nephron.

[34] Almutairi FM, Al-Duais MA, Shalaby KA, Sakran MI (2017). Analysis of patients with end-stage renal disease on dialysis in Tabuk City, Saudi Arabia: A single-center, three-year retrospective study. Saudi J Kidney Dis Transpl..

[35] Althumiri NA, Basyouni MH, AlMousa N, AlJuwaysim MF, Almubark RA, BinDhim NF (2021). Obesity in Saudi Arabia in 2020: prevalence, distribution, and its current association with various health conditions. Healthcare (Basel).

[36] ANZDATA 46th Annual Report 2023 (data to 2022). ANZDATA 46th Annual Report 2023 . November 23, 2023. Accessed 21 Dec 2023. https://www.anzdata.org.au/report/anzdata-46th-annual-report-2023-data-to-2022/.

[37] Venuthurupalli SK, Healy H, Fassett R, Cameron A, Wang Z, Hoy WE (2019). Chronic kidney disease, Queensland: Profile of patients with chronic kidney disease from regional Queensland, Australia: A registry report. Nephrology (Carlton).

[38] Venuthurupalli SK, Hoy WE, Healy HG, Cameron A, Fassett RG. CKD.QLD: establishment of a chronic kidney disease [CKD] registry in Queensland, Australia. BMC Nephrol. 2017;18(1):189. Published 2017 Jun 7. 10.1186/s12882-017-0607-5. 10.1186/s12882-017-0607-5 PMC5463396 28592254

