# Supplementary material for: Establishing the Kidney dIsease in the National guarD (KIND) registry: an opportunity for epidemiological and clinical research in Saudi Arabia
Source: BMC Nephrol. 2024 Feb 19;25:59. doi: 10.1186/s12882-024-03479-0 (PMC10875783; doi:10.1186/s12882-024-03479-0)
Supplement: Supplementary file 1 — Additional file 1: Supplementary Table 1. Results of renal biopsy if biopsy procedure was performed (n=587). [file 12882_2024_3479_MOESM1_ESM.docx]

Supplementary Table 1. Results of renal biopsy if biopsy procedure was performed (n=587).

| **Biopsy diagnosis** | **n** | **%** |
| --- | --- | --- |
| **Transplant biopsies** | **100** | **17.03** |
| 1. Acute T-cell rejection | 60 | 10.22 |
| 1. Chronic antibody-mediated rejection | 16 | 2.72 |
| 1. Mixed rejection | 10 | 1.7 |
| 1. Acute antibody-mediated rejection | 7 | 1.19 |
| 1. Calcineurin inhibitor toxicity | 4 | 0.68 |
| 1. Thrombotic microangiopathy | 2 | 0.34 |
| 1. BK nephropathy | 1 | 0.17 |
| **Glomerular diseases** | **336** | **57.24** |
| 1. Membranoproliferative glomerulonephritis | 151 | 25.72 |
| 1. Focal segmental glomerulosclerosis | 115 | 19.59 |
| 1. IgA nephropathy | 41 | 6.98 |
| 1. Lupus nephritis | 14 | 2.39 |
| 1. Minimal change disease | 10 | 1.7 |
| 1. ANCA-associated vasculitis | 2 | 0.34 |
| 1. Membranous nephropathy | 2 | 0.34 |
| 1. Amyloidosis | 1 | 0.17 |
| **Tubulointerstitial kidney disease** | **80** | **13.62** |
| **Diabetic kidney disease** | **48** | **8.18** |
| **Hypertension arterio-nephrosclerosis** | **8** | **1.36** |
| **Pyelonephritis** | **2** | **0.34** |
| **Other^*^** | **13** | **2.21** |

^*^Other= The biopsy sample was either nondiagnostic or inadequate to make a diagnosis
ANCA: Antineutrophilic cytoplasmic antibody; IgA: immunoglobulin A.
